# Mapping the purple menace: spatiotemporal distribution of purple loosestrife (*Lythrum salicaria*) along roadsides in northern New York State

**DOI:** 10.1038/s41598-022-09194-w

**Published:** 2022-03-28

**Authors:** Jessica Rogers, Kamal Humagain, Adam Pearson

**Affiliations:** 1 Department of Environmental Studies, State University of New York at Potsdam, Potsdam, USA; 2 Department of Geology, State University of New York at Potsdam, Potsdam, USA

**Keywords:** Invasive species, Restoration ecology, Wetlands ecology, Invasive species, Wetlands ecology

## Abstract

Purple loosestrife (*Lythrum salicaria L.*) is an invasive, herbaceous plant, frequently found in wetlands, creating monoculture stands, resulting in intensive management strategies in central New York, Ontario, and Quebec. The goal of this study was to identify the extent of infestations and to investigate factors that promote the spread of purple loosestrife. We attempted to answer several questions regarding level of infestation, connection to mowing, and influence of culverts. During flowering season in July and August, 2017–2019, we mapped infestations along 150 km (93 miles) of state highway between the Adirondack Park and the St. Lawrence River using the ESRI Collector app. The results of our preliminary analysis revealed significant increase in the number of plants (*P* < 0.001). In addition, a linear correlation analysis demonstrated a higher loosestrife density with an increase in plant species richness and a decrease in the distance to the closest infestation and wetland (*P* < 0.001 each). We found no statistical evidence that mowing promotes the spread of loosestrife. As expected, there were more individual infestations in highway ditches, but larger and denser infestations in wetlands (*P* = 0.003 in 2019). Culverts enable purple loosestrife to spread underneath highways and should be managed to prevent spread.

## Introduction

Roadways often provide semi-disturbed habitat for both native and non-native plant species to thrive^1^, as well as providing a corridor for invasive plants to spread throughout a landscape^2,3^. Roads can cut through ecosystems and create openings for new invasive species to enter a naïve system^3^. Lazaro-Lobo & Ervin^1^ did a comprehensive survey of over a thousand studies on the impact of roads on native vs non-native species, concluding that roadside verges provide habitat and a pathway for dispersal for both native and non-native species. To prevent non-native species taking over the roadsides, careful management is required to promote desired plant assemblages.

In addition to the roadsides themselves creating habitat, the traffic that travels along roadways can impact the vegetation growing along them. The increased wind from high-trafficked roads can increase the spread of seeds, specifically increasing dispersal distance^4^. Lembke et al.^4^ found that wind created on roads can increase wind-dispersed seeds, such as ragweed, along the roadsides, with a positive correlation with traffic volume. Road maintenance, such as grading unpaved roads^5^, paving or re-paving roads^6^, or mowing and even snowplowing, can move seeds or propagules longer distances than the plant’s own dispersion would generally suggest. Like unwashed boats moving aquatic invasive species between lakes^7^, mowing and road maintenance machinery can retain seeds or cuttings and relocate plants from one location to another, in addition to spreading laterally along the route^5,8^.

Multiple directed studies have been done to examine the spread of specific invasive plants along roadsides that cause damage to ecosystems or humans. Studies of the common reed (*Phragmites australis* (Cav.) Trin. ex Steud.) demonstrated that the invasive variety spread inland from the initial infestation along the St. Lawrence River, particularly quickly as a result of the highway system^9^. Common ragweed (*Ambrosia artemisiifolia* L.), the scourge of allergy sufferers, is most commonly found along paved roads and highways, likely as a conduit to invading agricultural fields^10^. In addition, Lemke et al.^4^ found that traffic along roads increases the spread of common ragweed. The traffic increased the spread of seeds by an order of magnitude, specifically in the driving direction.

This study focused on purple loosestrife (*Lythrum salicaria* L.), an invasive herbaceous plant of Eurasian origin^11^, which has been found in North America since the early 1800s^12^. It was sighted specifically in New York State and was likely found throughout southern Ontario and northern New York prior to the 1940s. Since the early 1990s, this species’ infestations have been well documented in central New York, specifically along the New York State Thruway^13,14^. There have been similar recordings of infestations in Ontario and Quebec^15^. As of 2011, purple loosestrife was one of the four priority species for the New York State Department of Transportation to focus on along roadsides (in addition to common reed (*Phragmites australis* (Cav.) Trin. ex Steud.), Japanese knotweed (*Polgonum cupidatum* Siebold & Zucc.), and Giant Hogweed (*Heracleum mantegassianum* Sommier & Levier))^16^. Great effort has been put into studying purple loosestrife’s natural history and to promote its control in central New York beginning in the 1990s^11,17–19^. Further studies aimed to evaluate successful management of biological controls in New York^20^ as well as in Ontario and Quebec^15,21^. However, very little monitoring has been done in between these larger studied populations along the New York side of the St. Lawrence River and inland toward the Adirondack Park. Purple loosestrife likely spread from both populations as the St. Lawrence River system flows throughout our study area, with tributaries draining from both the north and south, but genetic analysis would be required to confirm this. Purple loosestrife has been prevalent in several wetlands throughout the study area for between 15 and 20 years, as older images of wetlands show very minimal or no presence of invasions, and there are still many uninvaded areas. Currently, purple loosestrife is listed as a noxious weed in 38 states and is present in at least 45 of them^22^.

Purple loosestrife grows to 1–2 m tall, with 30–50 stems creating a candelabra effect from a single rootstock^11^. These stems are capable of casting hundreds of thousands of seeds, creating a very robust seed bank^23^. Mature stands can disperse seeds predominantly through water, with occasional, short distance (> 10 m) spread by wind, with dense stands forming after 4–5 years^11^. Seeds will germinate in most moist soils, across a gradient of soil types, and can dominate a freshwater wetland, even under various levels of pollution stresses^11^. In greenhouse settings, cuttings of 5–15 cm of purple loosestrife have also been shown to produce adventitious roots and lateral growth^24^.

There is some debate about the short-term and long-term effects of an invasion of purple loosestrife creating monocultures of non-native wetland plants and limiting biodiversity in wetlands^11,25,26^. Additional studies have examined the effect of purple loosestrife on the diversity of plants, specifically in wetlands, and found little or no decline in diversity^27^ while further studies found an increase in wetland diversity in the presence of purple loosestrife^28^. Overall, the consensus is that purple loosestrife has limited value in the ecosystem as a source of nutrients or habitat for wildlife and disrupts a functional ecosystem^29^. The impact of purple loosestrife on soil nutrients has been well documented, describing the change in available nutrients for other wetland plants, specifically cattails (*Typha latifolia L.*)^30^. Part of this uncertainty about long-term impacts involves the lack of information about the species’ rate of spread. While its seed production and the seeds’ ability to germinate in a variety of environments present clear advantages over native species^13^, the rate of spread outside a controlled environment, even over a short time, has not been well studied.

Roadsides present an avenue that purple loosestrife takes advantage of, due to its seeds’ ability to germinate in ephemeral water sources and in a variety of soil types. Mowing the verges of roadsides is a regular occurrence and has an impact on the plant species that live there. Studies have demonstrated that purple loosestrife is capable of creating adventitious roots and lateral growth from 5 to 15 cm cuttings, which is what would be created by mowing to keep roadsides clear^24^. In addition, mowing after seeds have set could spread seeds and small propagules to new areas carried by machinery^5^. Mowing can be used to control various invasive plant populations, such as reducing the growth of large-leafed lupine (*Lupinus polyphyllus* Lindl.)^31^ or mowing ragweed before pollen is released could reduce the population as well as mitigate allergens^32^. However, in the case of ragweed, mowing may be spreading the seeds as well^33^. It is not clear if a single mowing could affect all the different species suggested for control by mowing or if repeated mowing in a single season is required. The local NYS Department of Transportation (DOT) reported that they mow the roadsides of the entire region at least once per summer all the way to the edge of the right of way (~ 16 ft) and attempt to mow at least the minimal area (~ 8 ft) a second time. The frequency and timing of mowing is based on staffing, weather, and sufficient machine availability (James Ayers NYS DOT, personal communication).

In addition to mowing as a mechanism for spread, culverts exist under roadways to provide habitat connectivity, and allow water flow^34^, but can also be avenues for invasive species to spread from drainage ditches on either side of the road, allowing both native and invasive species to spread^35^. As restoration ecology has promoted habitat connectivity, to restore the movement of aquatic species, particularly fish, it is necessary to understand how invasive species might take advantage of that same restoration to spread into new environments^35^. Purple loosestrife seeds can travel in water^13^ and the ephemeral nature of water flowing through roadside drainage ditches would create moist soils ideal for future germination^11^.

The goal of this study was to document every occurrence, described as an infestation, of purple loosestrife in an under-studied area between two larger infested areas in central New York and across the St. Lawrence River in Ontario and Quebec. The lack of previous data describing the level of infestation has led to minimal management by local environmental protection agencies and has potentially allowed an invasive plant to spread unchecked throughout a naïve system. Once the initial mapping was completed, there were several questions that emerged about the annual spread and increase in the number of plants, methods for limiting the spread, and future management options. Because mowing can be used both to control roadside invasive plants^30–32^ and to spread invasive plants such as ragweed^33^, we wanted to investigate if mowing by the state or county was contributing to the spread of purple loosestrife by mapping all existing infestations over the course of several years.

In addition, we wanted to know if the presence of culverts beneath the roads was contributing to the spread. Our hope was to find sources of spread that could be the target of future management actions. Over the course of 3 years, during plant flowering season in late July and August, we mapped all infestations of purple loosestrife in addition to all of their co-habitant plant species, along 93 miles of state highways in St. Lawrence and Jefferson counties in northern New York State.

## Results

### Overall increase in infestation and density

After 3 years of data collection from 2017 to 2019, we found many more individual infestations than expected or had been previously documented. There were 663 individual infestations in 2017, with an average roadside length of 42.6 m, with an average number of 74 plants (range 1–3100 plants). However, nearly 24% of infestations (n = 158) only had one plant. For 2018, there were 685 individual infestations, and the average roadside length continued to be 42.6 m, but the average number of plants increased to 82 plants (range 1–3000 plants), but only 10% of infestations had a single plant. In 2019, the number of individual infestations decreased to 537 infestations. However, the average roadside length of an infestation increased to 69.3 m, and 158 plants (range 1–3500 plants), and only 8% of infestations had only a single plant (See Table 1).

**Table 1:** Descriptions of infestations across the study time frame.

Year of data collection	2017	2018	2019
Number of infestations	663	685	537
Total number of plants	48,901	56,215	85,152
Total length of route infested (km)	28.2 km	29.0 km	36.17 km
Mean/Median size of infestation (range)	42.6 m/12 m (1–1600 m)	42.6 m/14.5 m (140 ft)/(1–743 m)	69.3 m/24.3 m (1–978 m)
Mean/Median number of plants (range)	74/10 plants (1–3100)	82.4/15 plants (1–3000)	158.6/25 plants (1–3500)
Mean/Median density	1.63/1.00 (plants/m)	2.02/1.30 (plants/m)	4.70/1.43 (plants/m)
Infestations with a single plant	24%	10%	8%

In addition to examining the differences in sizes of infestations and the total number of plants, we examined the changes in density from year to year. There was a significant increase in density between 2017 and 2018 (*P* = 0.001), but not between 2018 and 2019 (*P* = 0.459), but over the whole time period there also was a significant increase (*P* = 0.002).

We compared the summarized polygons across years to investigate the change in the total roadside length infestation within each polygon and the total number of plants within each polygon over time. We note that all summarized polygons are equal in size and therefore number of plants within a polygon is equal to its density within the same polygon. The results show significant increases from 2018 to 2019 (*P* = 0.002) and over the entire time from 2017 to 2019 (*P* < 0.001). The change from 2017 to 2018 did not show a significant increase in total roadside length infestations or number of plants (*P* = 0.12)*.* In total, there was a 14% increase in the number of plants from 2017 to 2018, and a 51% increase from 2018 to 2019.

### Water type, wetlands, and culverts

**Figure 1:**
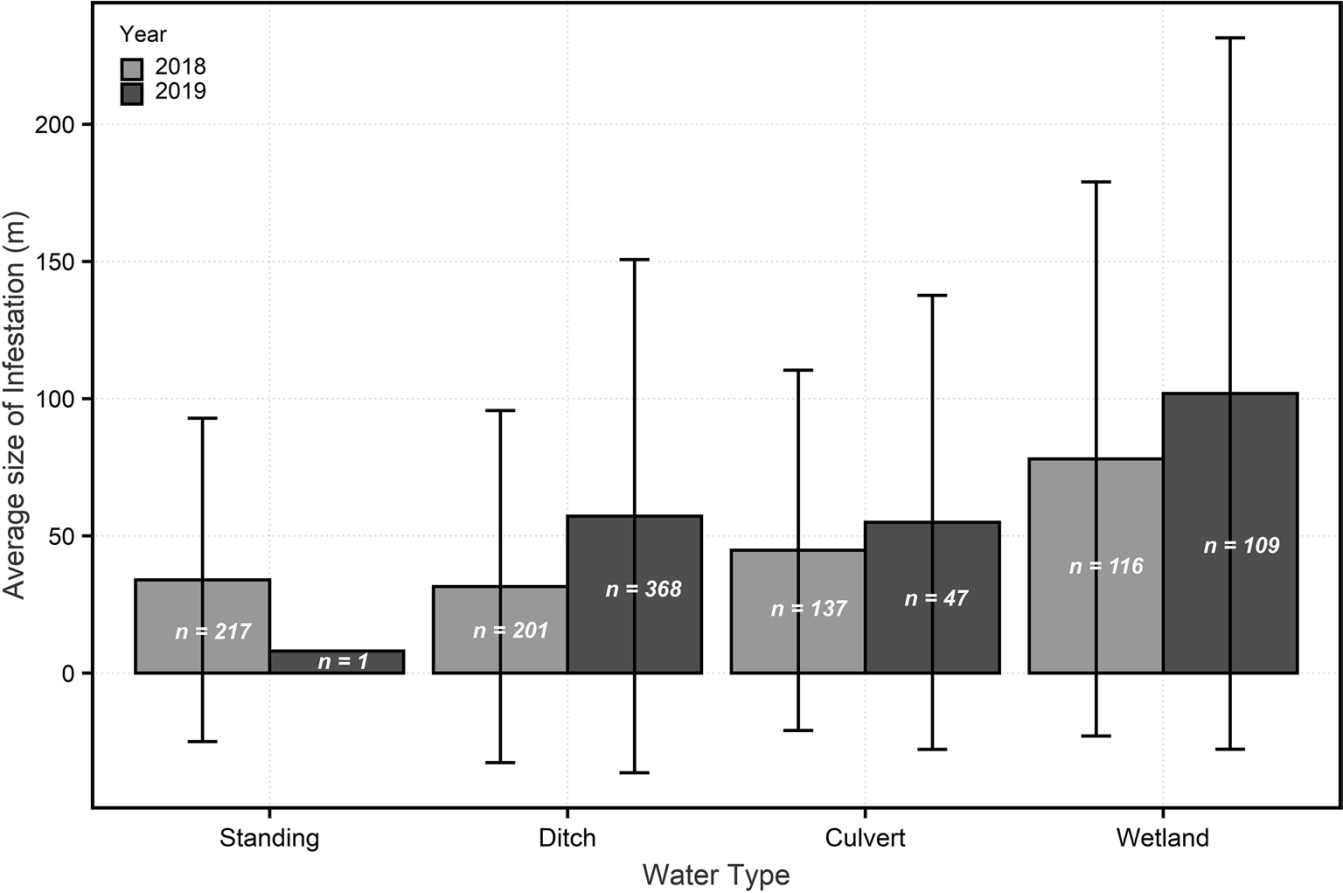
Size of infestations by water type.

During data collection in 2017, we developed the definitions of water type categories and created consistency. Therefore, 2017 was not used for analysis of differences in infestation size by water type. In 2018, there was a significant difference between infestations sizes by water type (*P* = 0.002), and again in 2019 (*P* < 0.001). The largest infestations were found in wetlands, though wetlands only made up ~ 20% of the infestations, while ditches and standing water made up the predominant number of infestations. Both ditches and standing water had more individual infestations than wetlands (Fig. 1). Across all years, there was a significant difference in density by water type (*P* = 0.004).

**Figure 2:**
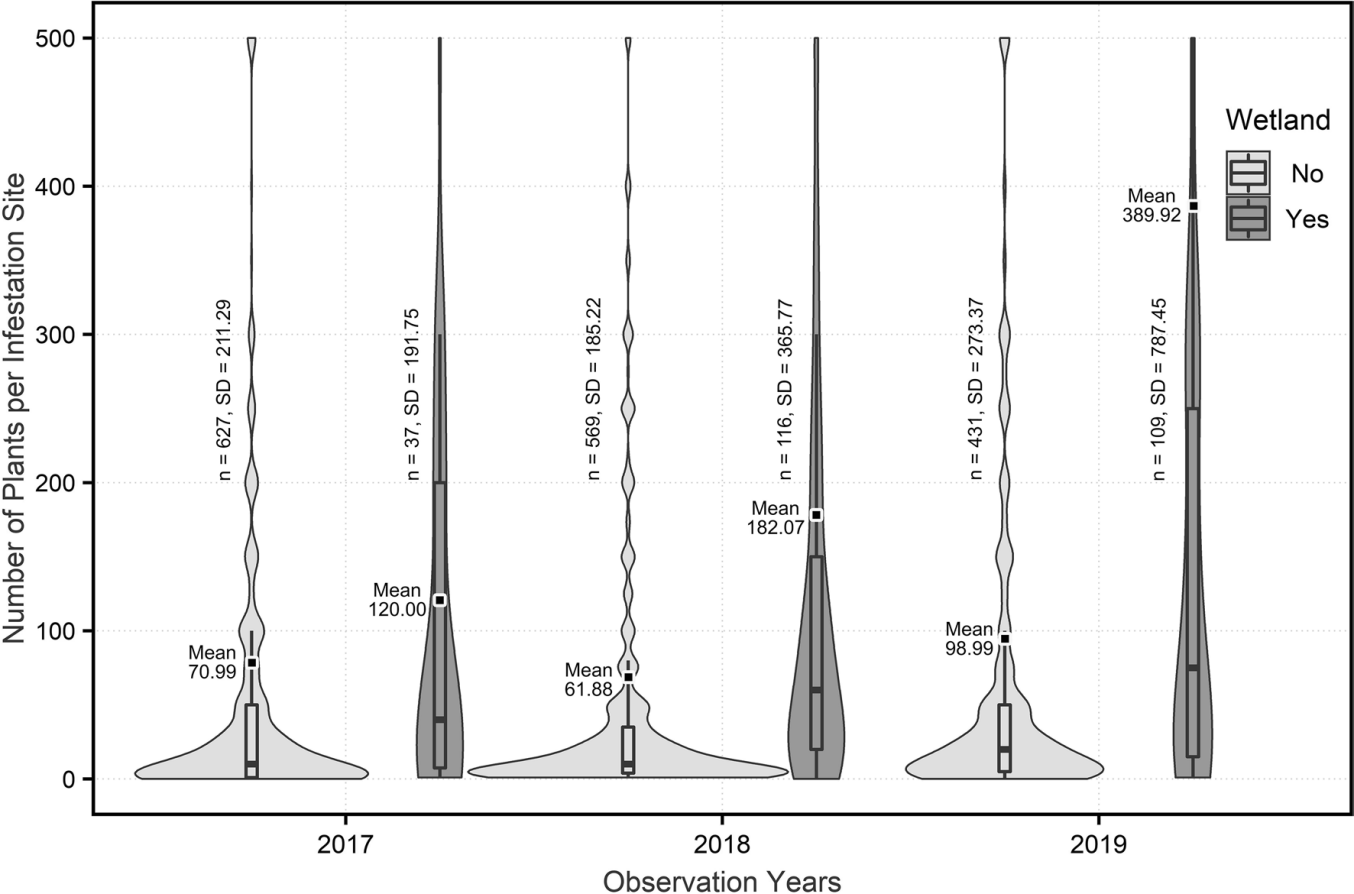
Annual changes in number of plants inside wetland areas and outside wetland areas (Wider areas represent higher number of infestation sites, n = number of observations, SD = Standard Deviation, box plots represent medians and quartiles, and the dots represent means). Figure created in RStudio v9.1.372^36^ using R v4.1.2^37^.

The increase in number of plants and size of infestations was found predominantly in wetlands, compared to outside a wetland. Because the data was not normally distributed, a non-parametric Mann–Whitney U test demonstrated that in 2018 and 2019, there was a significantly higher number of plants in wetland infestations than outside wetlands (*P* = 0.001 and *P* = 0.003 respectively) (Fig. 2). There was no significant trend in 2017 (*P* = 0.077).

Comparing culverts to random points demonstrated a strong influence of culverts on the presence and number of purple loosestrife plants. A two-samples chi-square analysis revealed significantly more purple loosestrife plants at culverts compared to random points (*P* < 0.001). Understanding that culverts are generally placed where known water passes under roadways, and might simply create habitat for purple loosestrife, we examined all 91 areas where wetlands are split by a road^38^. We found that while wetlands are the favored habitat for purple loosestrife and culverts are often placed in wetlands, in areas with known water and a culvert, 66% of those had purple loosestrife on both sides of the road, compared to only 28% of road-crossing wetlands without a culvert. This suggests that our initial comparison of culverts with random points is supported even when controlled for wetland habitats.

### Directional spread

We found no significance from the McNemar test (*P* = 0.686) that mowing is causing a single directional infestation to spread.

### Multiple linear regression analysis

**Figure 3:**
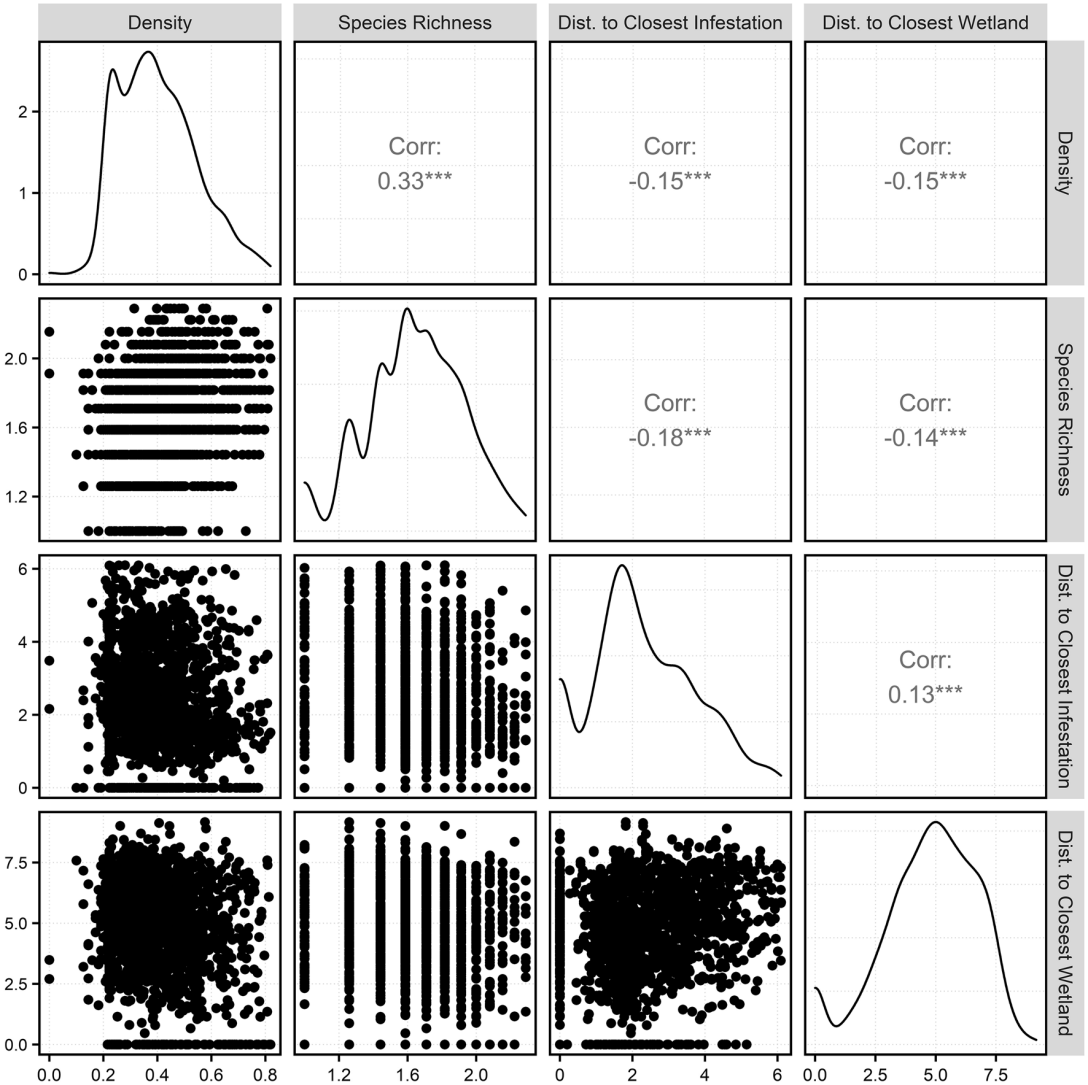
Pairwise plots to compare the purple loosestrife density (number of plants/m^2^), plant species richness (number of species), distance to closest infestation (m), and distance to closest wetland (m). Cube root transformation was implemented for all the variables used in the model to normalize the data. Diagonal plots represent the general distribution curve demonstrating normality and the values in the plot represent correlation between the variables. Figure created in RStudio v9.1.372^36^ using R v4.1.2^37^.

Based on the cube root transformation of the response (plant density) and predictor variables (accompanying plant species richness, distance to the nearest infestation, and distance to the nearest wetland), we found a positive correlation between the density and plant species richness (0.34) (Fig. 3). In contrast, the distance to the closet infestation and the distance to the nearest wetland were negatively correlated with the density (− 0.15 each; Fig. 3).

Using multiple linear regression for the 3 years of observations (n = 1673), we found significant relationships between the plant density and distance to the closest wetland, distance to the closest infestation, and plant species richness (*P* < 0.001 each; Table 2). According to the model (*F*_3,1669_ = 82.67, *P* < 0.001), higher densities of purple loosestrife is associated with higher species richness and lower distances to the closest infestation and wetland. Even though about 13% of the variability is explained by the model (*R*^*2*^ = 0.1294, adjusted *R*^*2*^ = 0.1278), the predictor variables are significant at *P* < 0.001. To validate the model, we created diagnostic plots and tenfold cross validation. Diagnostic plots demonstrate the normal distribution of the residuals of the regression model (see Supplemental Material). Based on the tenfold cross validation, we found a root mean square error of 0.1315 and mean absolute error of 0.1059.

**Table 2:** Multiple Linear Regression model to predict density (number of plants/m^2^ ) using plant species richness (number of plant species), distance to the closest infestation (m), and distance to the closest wetland (m) as predictor variables (n = 1673).

Variables	Estimate	Standard error	*P*-value	Mean
Intercept	0.2030	0.0230	< 0.001	
Species richness	0.1528	0.0116	< 0.001	1.6440
Distance to infestation	− 0.0080	0.0023	< 0.001	2.3002
Distance to wetlands	− 0.0071	0.0017	< 0.001	4.7571

Cube root transformation was implemented for all the variables used in the model to normalize the data.

## Discussion

Over the 3 years of the study the total number of plants increased, while the number of infestations decreased. Based on visual inspection of the detailed maps, the fewer infestations were likely due to previously smaller infestations connecting and becoming larger infestations in successive years. The downward trend in the number of infestations with a single plant supports this idea. The increase in the number of plants is consistent with the strong ability of purple loosestrife to produce thousands and thousands of seeds^11^. Yakimowski et al.^39^ also found that the seed bank within invaded wetlands contained enormous numbers of purple loosestrife seeds (several hundred thousand/m^2^) and suggested that only environmental variations changed the emergence of seeds within an invaded wetland. Inconsistent weather patterns between years in our study and a lack of soil moisture data leave us unable to determine the connection between rainfall and germination in our study area.

Infestations were larger and had higher density inside wetlands along roads compared to other habitats along roadsides, which is to be expected given the preference purple loosestrife has for germinating seeds in moist soil after being dispersed by water^11^. The ephemeral nature of water in ditches alongside the road has allowed the infestations to sustain themselves and grow larger in wetter years given their likely substantial seedbank^23^. The concern is the spread in small numbers along roadsides in ditches connecting wetlands will lead to invasions in all wetlands over time.

Further, the width of the road should be an impediment to the natural spread of purple loosestrife, as its seeds rarely spread independently beyond 10 m^11^. However, culverts that carry water under the road create another mechanism for spreading purple loosestrife between wetlands. We found that purple loosestrife infestations were found more frequently at culverts and more frequently on both sides of the road next to culverts compared to random sites along the route. This suggests that culverts are increasing the spread, potentially into new areas. In particular, culverts at wetlands are potentially increasing the spread into areas on the other side of the road, compared to wetlands without culverts. Wilcox^14^ also found that culverts under the New York State Thruway increased the spread of purple loosestrife along the highway, rather than from sites outside the road corridor. The assumed movement along ditches in times of higher water would also confirm the evidence that purple loosestrife is moving through culverts to spread to both sides of the roads. Understanding that culverts are generally placed where known water passes under roadways, and might simply create habitat for purple loosestrife, we examined all 91 areas where wetlands or streams exist on both sides of the road or are split by the road^38^. We found that while wetlands are the favored habitat for purple loosestrife and culverts are often placed in wetlands, in areas with known water and a culvert 66% of those had purple loosestrife on both sides of the road, compared to only 28% of areas with water water but without a culvert. This suggests that our initial comparison of culverts with random points is supported even when controlled for wetland habitats.

The analysis of adjacent polygons failed to reveal a directional spread of purple loosestrife due to mowing, despite Wilcox^14^ demonstrating that adjacent polygons containing infestations increased the likelihood of infestation. Our original goal to determine if mowing along the highway was promoting the spread of purple loosestrife was inconclusive. This could be due to the relatively large size of the polygons in the analysis compared to very small infestations. It was not practical to examine individual infestations and estimate spread from year to year to capture individual population spreading. It is likely that water availability and proximity to wetlands more strongly affected the spread of purple loosestrife than mowing in our region. As wetlands were spaced throughout the study area (71 of 186 polygons contained wetlands), each was providing a source for infestations to increase in density and likely to spread out from that location based on movements of water (e.g., culverts or ditches) rather than solely by being mowed. In addition, the local NYS DOT office (James Ayers, personal communication Mar 2021) described the patterns of mowing as twice each summer spread throughout the region, so each area gets mowed at slightly different times of the summer. For example, it is possible that regions with high infestations have previously been mowed when the seeds were ready to spread, while in other parts of the study, mowing might have been done prior to seed being set. We recommend actions that avoid the spread to uninvaded wetlands. The substantial seeds throughout infested wetlands have been previously reported, even in parts of the wetland without obvious plant growth^39^.

Thomas and Moloney^40^ predicted invasions of wetlands based on local landcover variation. While their study was predominantly in an urban area centered around St. Paul/Minneapolis, the variations of land use have implications for our study. The study area is a mixture of lawns and state-mowed roadsides and creates a varied landscape where the spread of purple loosestrife is stopped by lawns, given the more frequent mowing and care landowners give their cultivated lawns. Finer scale analysis and the inclusion of individual lawns as possible breaks in spread could reveal the connection between mowing and spread of purple loosestrife.

While controlling spread through mowing is one strategy, controlling the overall infestation with other management strategies, including biological controls, has had some success and has been well studied^21,41,42^. We did track the presence of herbivory across the study area and found some clusters of likely areas with beetles, though no beetles were seen in the first 2 years of the study (beetles emerge and generally complete their life cycle prior to the emergence of inflorescence). In 2018, we began to release *Galerucella sp.* beetles at one of the wetlands and have been monitoring these sites biannually since. In 2019, we propagated several thousand *Galerucella calmariensis* L. beetles at SUNY Potsdam according to methods by Rowell^43^. We intend to continue monitoring any released beetles at the largest infestations using techniques developed by Dr. Bernd Blossey at Cornell University (personal communication).

The spatiotemporal analysis of the infestations did reveal several results by examining various spatial variables, such as slope, distance to nearest infestation, distance to nearest wetland, and the connection to species richness around an infestation. Given the dominance of purple loosestrife in wetlands, as described above, it is unsurprising that the distance to wetlands was negatively correlated to increased species density in the linear regression model. Higher densities of plants are found closer to wetlands. In addition, higher densities of purple loosestrife were found in closer proximity to other infestations, which does contribute to the concept that species can be spread from mowing, though it doesn’t necessarily distinguish mowing as the cause beyond the plant’s innate ability to spread.

Most revealing, and worth further analysis in the future, is the correlation between the increased purple loosestrife density and increased species richness. All plant species within 1 m of a purple loosestrife infestation were recorded, and increased species richness was a predictor of higher purple loosestrife density. Species richness and diversity along roadside right of way (ROW) has been studied as it connects to the spread from the ROW to neighboring forests or hedgerows^44^ or grasslands^45^, and agriculture landscapes^46^ as well as the conservation value of ROWs for avian and small mammal conservation^46^. These studies most often focus on the conservation value or use as habitat for wildlife. However, few studies documented variations in species richness along roadsides. There have been many studies specifically within wetlands looking at the connection between purple loosestrife and plant diversity^26–28^, but none of these looked at the purple loosestrife and plant species diversity along roadsides. The analysis in this study revealed an increased species richness found with the highest density infestations likely due to purple loosestrife’s ability to germinate across a range of habitats, particularly transitional habitats from the edges of roads to nearby wetlands. The variability in roadside habitats also provides more types of habitats for generalist plant invaders as well. Because this study was observational and not experimental, it is unclear whether other areas along roadsides with high levels of diversity without purple loosestrife exist, but that would be worthy of future study.

## Conclusion

The study area in northern New York is at as much risk of purple loosestrife invasion as more studied areas due to a highly connected series of wetlands in the 4 major drainage basins from the Adirondacks into the St. Lawrence River. We succeeded in documenting the presence and increase of purple loosestrife infestations throughout the area, focusing on state highways due to high traffic and disturbance, over a 3-year period. We have shown that along roadsides in relatively disturbed habitat, wetlands are where purple loosestrife is most prevalent; and culverts are enabling infestations to spread underneath roads which would normally be a natural barrier to purple loosestrife. In addition, increased plant species richness predicts higher density of purple loosestrife and understanding that connection outside of wetlands will be an important part of future studies. Left unmanaged, purple loosestrife will continue to infest the wetlands of northern New York and understanding each of the locations and knowing where to focus on mowing and management are crucial.

Our next steps turn towards investigating appropriate management options for the study area in order to seek to control the spread of purple loosestrife. Despite being unable to demonstrate an effect of directional spread via mowing, we will work carefully with the NYS Department of Transportation in our region to coordinate the timing of mowing to avoid coinciding with seed set in the largest infested areas or proximity to uninvaded wetlands. Given the DOT’s strong willingness to make changes based on evidence, it would be helpful to understand in more detail how these kinds of high-volume mowers spread purple loosestrife, building on previous work for other species. Reaching out to those landowners along the study route, particularly between wetlands, to stop the spread in smaller infestations, based on our findings, could have a big impact on minimizing the spread between wetlands.

Future management of purple loosestrife must include biological control. We have begun releasing *Galerucella* sp. beetles, first from captured beetles the NYS DEC provided from central New York, and then propagating our own in 2019 and 2021, to increase their presence in the area. This study was done before beetles were widely released throughout the area and no beetles were seen in our initial field seasons, though evidence of some herbivory was noted. Comparisons of these infestations to post-beetle release monitoring are crucial to understand when we have reached the threshold of ecosystem equilibrium. Several more years of beetle release and monitoring are necessary to understand its success in this ecosystem.

## Methods

### Study site

**Figure 4:**
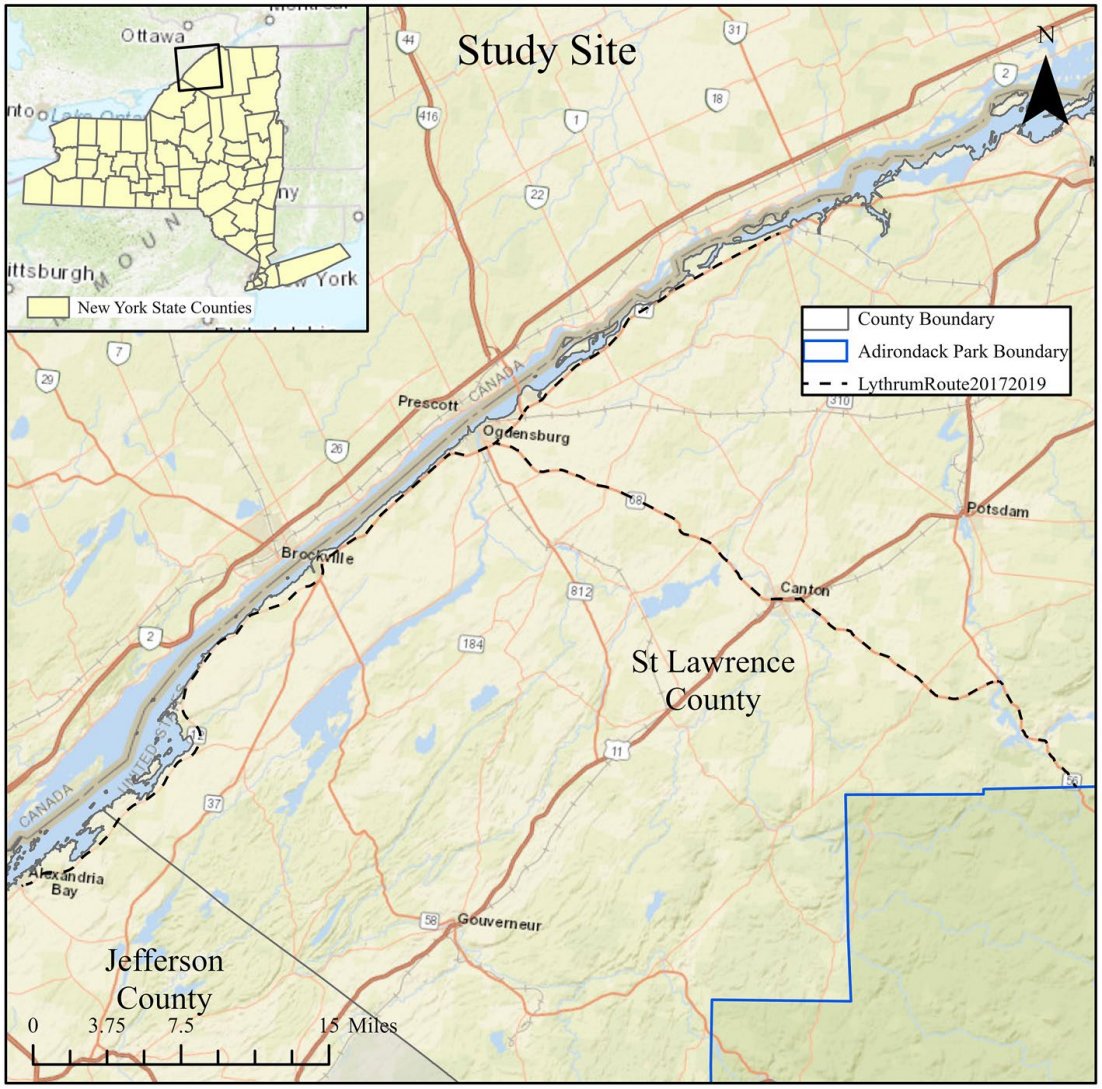
Map of the study site, created in ArcGIS Pro v2.9^50^. The study route followed State Route 56 from the Adirondack Park boundary, to State Route 68 in Colton to Ogdensburg, north along State Route 37 to Waddington, and south along State Route 37 to State Route 12 to Alexandria Bay.

The study area in Northern New York was chosen because initial informal surveys clearly indicated far greater numbers of infestations than the New York State Department of Environmental Conservation (NYS DEC) indicated on their website^47^. We contained our research to state highways in St. Lawrence and Jefferson Counties in northern New York (Fig. 4 and^48^ for detailed description of the study area). State highways were chosen as they represented the largest traffic volume spreading through the area from the Adirondack Park to the St. Lawrence River. New York State Department of Transportation manages all of the roads we followed, and permission was granted to allow us to walk along roadsides and collect data in their right of way, any area maintained as a clear zone along the highway^49^. There are no interstate highways in St. Lawrence County, and our data collection connected the major interstate highway to the west in Jefferson County, along the St. Lawrence River to the Adirondack Park. Management inside the Park is handled by specific state agencies and prioritizes environmental protection rarely seen outside a protected area.

### Data collection

Data were collected over 3 successive summer seasons from 2017 to 2019 using the Collector app from Environmental Systems Research Institute (ESRI) on mobile devices. A survey template was created with predetermined fields to limit variation from person to person and year to year. Undergraduate student research assistants were trained each summer in the identification of purple loosestrife and other herbaceous plants known to exist within the region. The first day of training involved practice with the ESRI Collector app and identifying plants, using plant identification books as well as plant identification apps (e.g., Picture This!). All observed purple loosestrife was counted along the entire 150 km(93 mile)-route. Individual infestations, defined as any growth of purple loosestrife plants, were distinguished by having no other purple loosestrife plants closer than 6 m. For all infestations under 25 plants, the plants were counted individually. Above 25 plants, estimates were performed using a 1 m^2^ quadrat to practice estimating the number of purple loosestrife plants by putting it into different densities of infestations and counting all the stems within the quadrat. All researchers practiced estimations until they could estimate infestations to a similar degree. Above 25 plants, estimates grew by increments of 25 up to 500 plants, and beyond that estimates grew by 100.

Individual infestations were distinguished if they occurred more than 6 m apart, and the infestation had to include at least one flowering plant to confirm plant identification. The data collected for each infestation included the date, time, Global Positioning System (GPS) location, collector’s name, distance from the highway (< 5 m), the length of the invasion along the road (m), the estimated number of plants, average height of the plants (m). We noted whether herbivory was present or absent, (in 2018 and 2019 we did estimate more than presence/absence of herbivory) indicated by holes in the leaves/petals or presence of insects. In 2017, we only noted the presence or absence of herbivory based on missing sections of leaves. In 2018 and 2019, based on more observations, we created a scale of level of herbivory from “No herbivory”, indicated by full leaves (less than 5% missing), “Some Plants/Herbivory” indicated some leaves had considerable herbivory within an infestation or some leaves across the infestation had evidence of herbivory, “Most Plants/Herbivory” indicated by increased number of leaves damaged (> 50%), and/or increased level of damage, “All Plants” indicating that greater than 90% of the leaves/plants in the infestation had evidence of herbivory. To record species richness, we noted all other species present within 1 m of infestations, identifying all we could down to at least family (Asteraceae was difficult to identify to species at that time), but most we identified to species. We recorded what type of mowing was being done (e.g., a lawn mown by landowners to the road, or the 2.5–5 m of mowing done by the Department of Transportation (DOT) or no mowing at all (very rare)). We noted the type of water infestations grew in (from 5 described categories beginning in 2018). Water types included wetlands (flooded areas containing other plants species known to inhabit wetlands), ditches (depressions with risen sides with ephemeral water), standing water (flooded areas with no clear wetland characteristics), culverts (passages under roadways that did not connect to a wetland), or none (no evidence of any water pooling). A minimum of one photo was taken at each location of purple loosestrife and was georeferenced by the software. Location data accuracy was at least 5 m based on the internal GPS units on our mobile devices. Beginning in 2018, we added a Bad Elf Global Navigation Satellite System (GNSS) Surveyor, connected by Bluetooth to increase data accuracy, generally to less than 2 m (6 ft), but distinguishing infestations at 6 m (20 ft) was maintained despite increased accuracy.

Beginning in the 3rd week of July as the plants began to bloom, researchers walked along the highway following the direction of traffic, mapping each infestation. In 2017, data was collected as a point in the field and converted to a line parallel to the highway in ArcGIS Online. In 2018 and 2019, data was collected in the field as line data. Researchers walked for several hours in one direction noting infestations, then crossed the highway and returned to the point they started. Data collection lasted until the entire 150 km highway was mapped, until mid-August. The final 2 days of research each year involved driving the entire route slowly to confirm all data points and ensure any later blooming infestations were included.

Culvert information was collected manually. The NYS Department of Transportation had a GIS layer of culverts, but it contained many fewer than anecdotal knowledge suggested. Culvert points were collected both during purple loosestrife collection and independently as a separate layer. A culvert point was collected at any location that had a roadside marker with a small yellow reflective rectangle, confirmed with a pipe or tunnel under the highway where water could pass through. While the size of the culvert, from 1 m to more than 5 m for larger bridge-sized passages was collected, we did not distinguish by size in the analysis as water could pass through all culverts given the proper conditions (e.g., sufficient rainfall). Wetland data was obtained from the National Wetlands Inventory for New York State and clipped to the study region^38^.

### Analysis

All spatial analysis was done in ArcGIS Pro v2.9 and statistical analysis was done in IBM SPSS Statistics v25^51^, R version 4.1.2^37^, and RStudio v 9.1.372^36^. Because the data were not normally distributed, to compare differences between years, we used either the Mann–Whitney U test when comparing 2 years, or the Kruskal–Wallis Test to compare all 3 years.

**Figure 5:**
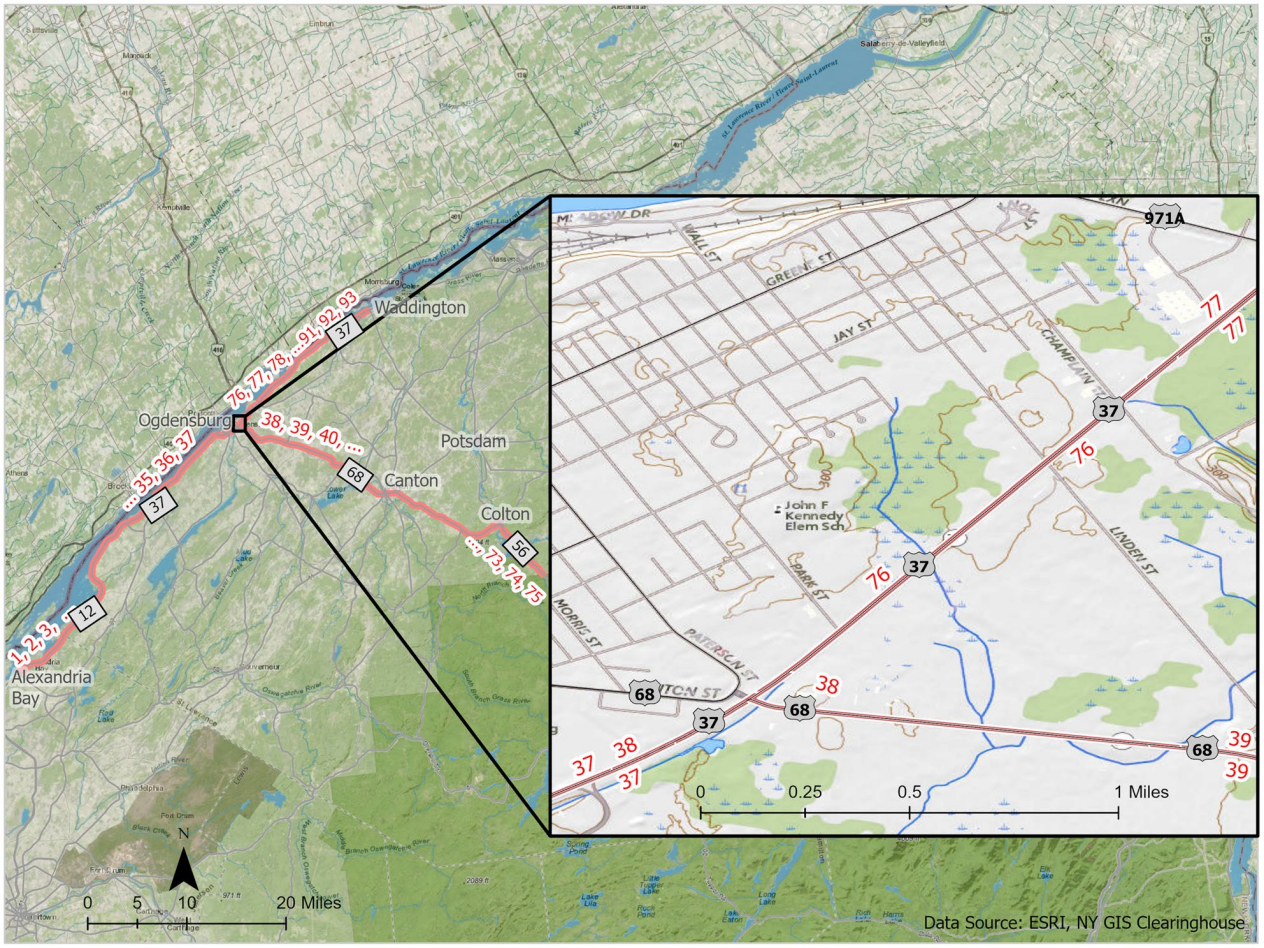
Map of polygons, inset of the junction of Routes 37 and 68, created in ArcGIS Pro v2.9^47^.

To capture changes to infestations, polygons were created along the entire route, each appropriately 1 mile in length (the meaningful measurement for the Department of Transportation) and 12 m from the center highway line. This created 93 polygons on each side of the road (186 total). Polygons were numbered beginning at the southwest end of the route in Alexandria Bay with Polygon 1, increasing to polygon 38 in Ogdensburg on Route 37. The route turned east to Route 68 and polygon 39, continuing to South Colton on Route 56, ending with polygon 75. Because there was extension of Route 37 to Waddington, the polygons continued numbering at 76, ending in Waddington with polygon 93. This meant that polygon 38 connected to both polygon 39, and polygon 76 (Fig. 5). This was noted for any analysis that examined spread.

The total size of infestations and the total number of plants within each polygon was summarized using the Summarize Within tool in ArcGIS Pro. To examine the connection between wetlands and infestations of purple loosestrife, the National Wetlands Inventory dataset^38^ was clipped to the study region and all wetlands within 5 m of a roadside polygon were included in the analysis examining the connection between the size of infestations and proximity to wetlands.

Each culvert point was manually examined and assigned to one of three categories: purple loosestrife on both sides, on only one side, or no purple loosestrife present. Random points were generated along the entire route using the Create Random Points tool in ArcGIS Pro. The random points were also assigned to one of the same 3 categories above. The culvert data and random data points were compared using a chi-squared statistical analysis to determine if culverts had a significant influence on increasing infestations.

We looked at each polygon and its neighbors (those numbered one less and one greater than each target polygon) to understand whether a directional spread could be detected. We hypothesized that the lower number polygon would have a greater influence on whether the target polygon had received its infestation as a result of directional infestation, due to mowing machines creating propagules and dragging seeds, compared to the higher number polygon. We assumed the prior polygon would have a greater influence because state and county roadside mowers always move in the direction of traffic, as did our numbering. If mowing caused directional infestation to spread, we would expect the target polygon to be populated and have an equal to or smaller infestation than the polygon immediately prior to it. In addition, we would expect the target polygon to be less influenced by the polygon immediately following it. We ran a McNemar test, following methods from Wilcox^14^ to look at spread of infestations.

In ArcGIS Pro, we created a 5-m buffer for the line data collected in the field. The resulting buffer polygons were used to calculate the density of the purple loosestrife plants per square meter. We ran multiple linear regression in R to predict the density using plant species richness, distance to the nearest infestation, and distance to the nearest wetland as predictor variables. The model included all years of data as individual data points after removing the outliers (n = 1673). We discarded the slope of the landscape from the model because it was not significant in predicting density. We used cube root transformation of the response and predictor variables to normalize the data. A tenfold cross validation method was used to validate the model that performed the fitting procedure 10 times with each fit performed on a randomly selected training set (90% of the data points) and test set (remaining 10%).

## Supplementary Information

Supplementary Information.
