# Supplementary material for: Mapping the purple menace: spatiotemporal distribution of purple loosestrife (Lythrum salicaria) along roadsides in northern New York State
Source: Sci Rep. 2022 Mar 28;12:5270. doi: 10.1038/s41598-022-09194-w (PMC8960840; doi:10.1038/s41598-022-09194-w)
Supplement: Supplementary file 1 — Supplementary Information. [file 41598_2022_9194_MOESM1_ESM.docx]

Supplemental Material for Rogers, Humagain, and Pearson

**Mapping the purple menace: Spatiotemporal distribution of Purple Loosestrife *(Lythrum salicaria*) along roadsides in northern New York State**

Below is the diagnostic plot used to validate the model, including the relevant paragraph from the original paper.

Using multiple linear regression for the three years of observations (n = 1673), we found significant relationships between the plant density and distance to the closest wetland, distance to the closest infestation, and plant species richness (*P* < 0.001 each; Table 2). According to the model (*F_3, 1669_*= 82.67, *P* < 0.001), higher densities of purple loosestrife is associated with higher species richness and lower distances to the closest infestation and wetland. Even though about 13% of the variability is explained by the model (*R^2^* = 0.1294, adjusted *R^2^* = 0.1278), the predictor variables are significant at *P <* 0.001. To validate the model, we created diagnostic plots and 10-fold cross validation. Diagnostic plots demonstrate the normal distribution of the residuals of the regression model (see Supplemental Material). Based on the 10-fold cross validation, we found a root mean square error of 0.1315 and mean absolute error of 0.1059.


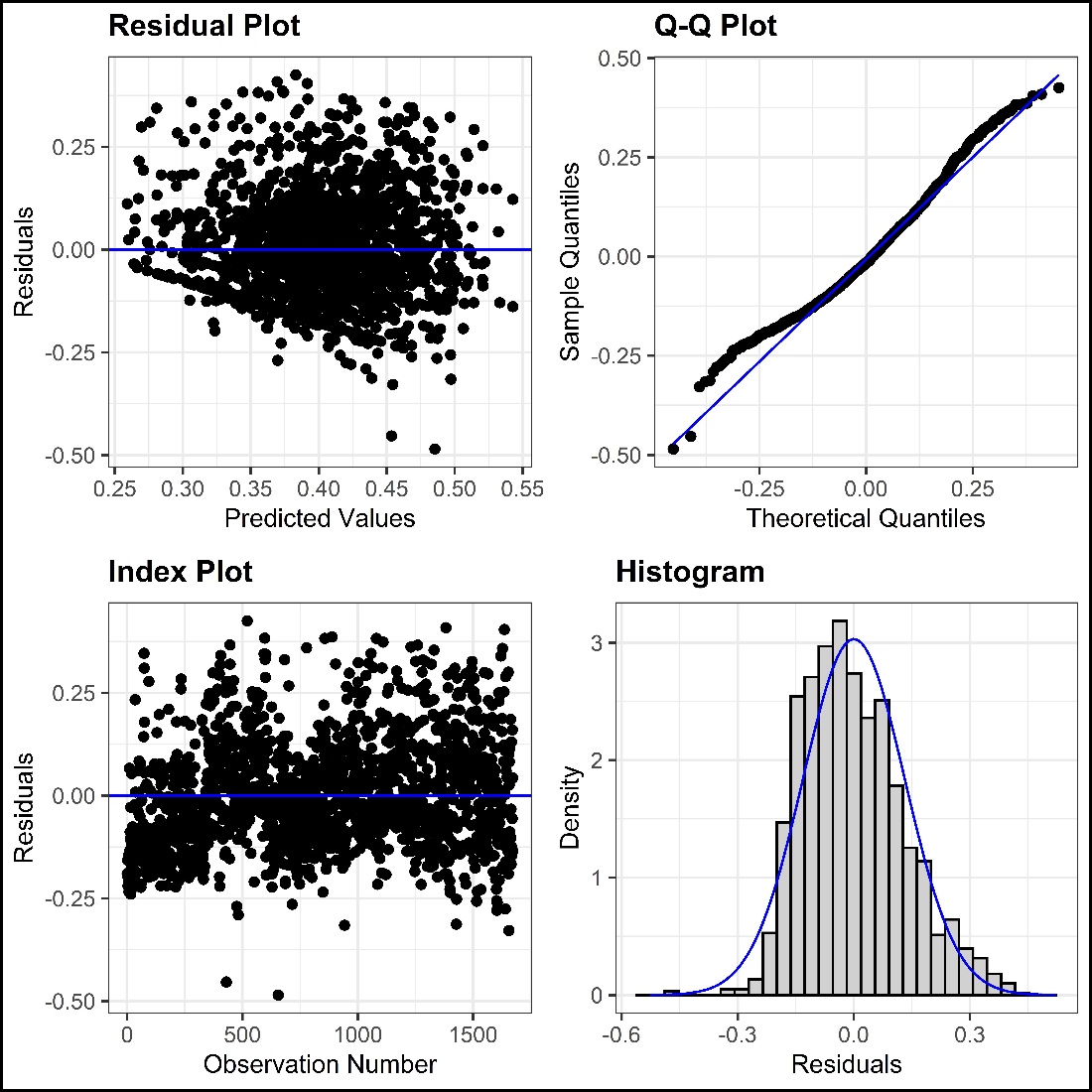


Supplemental Figure S1 - Diagnostic plots of the multiple linear regression model to predict density of purple loosestrife using plant species richness, distance to the nearest infestation, and distance to the nearest wetland. The model uses cube root transformation of the response variable and predictor variables. Figure created in RStudio v9.1.372 [34] using R v4.1.2 [35]
